# Exosomal ncRNAs in reproductive cancers^†^

**DOI:** 10.1093/biolre/ioae170

**Published:** 2024-11-19

**Authors:** Alicja Kowalczyk, Marcjanna Wrzecińska, Elżbieta Gałęska, Ewa Czerniawska-Piątkowska, Mercedes Camiña, Jose P Araujo, Zbigniew Dobrzański

**Affiliations:** Department of Environment Hygiene and Animal Welfare, Wrocław University of Environmental and Life Sciences, Wrocław, Poland; Department of Ruminant Science, West Pomeranian University of Technology in Szczecin, Szczecin, Poland; Department of Environment Hygiene and Animal Welfare, Wrocław University of Environmental and Life Sciences, Wrocław, Poland; Department of Ruminant Science, West Pomeranian University of Technology in Szczecin, Szczecin, Poland; Department of Physiology, University of Santiago de Compostela, Santiago de Compostela, Spain; Mountain Research Centre (CIMO), Instituto Politécnico de Viana do Castelo, Ponte de Lima, Portugal; Department of Environment Hygiene and Animal Welfare, Wrocław University of Environmental and Life Sciences, Wrocław, Poland

**Keywords:** extracellular vesicles, exosomes, cancers, reproductive cancers, microRNA, reproduction

## Abstract

Extracellular vesicles, particularly exosomes, play a pivotal role in the cellular mechanisms underlying cancer. This review explores the various functions of exosomes in the progression, growth, and metastasis of cancers affecting the male and female reproductive systems. Exosomes are identified as key mediators in intercellular communication, capable of transferring bioactive molecules such as microRNAs, proteins, and other nucleic acids that influence cancer cell behavior and tumor microenvironment interactions. It has been shown that non-coding RNAs transported by exosomes play an important role in tumor growth processes. Significant molecules that may serve as biomarkers in the development and progression of male reproductive cancers include miR-125a-5p, miR-21, miR-375, the miR-371 ~ 373 cluster, and miR-145-5p. For female reproductive cancers, significant microRNAs include miR-26a-5p, miR-148b, miR-205, and miRNA-423-3p. This review highlights the potential of these noncoding RNAs as biomarkers and prognostics in tumor diagnostics. Understanding the diverse roles of exosomes may hold promise for developing new therapeutic strategies and improving treatment outcomes for cancer patients.

## Teaser

**Summary Sentence**

Research into exosomal ncRNAs in reproductive cancers is crucial because it combines fundamental biological insights with immediate clinical applications. It holds the potential to revolutionize early detection, treatment personalization, and the understanding of metastatic and resistance mechanisms, ultimately improving outcomes for patients with these challenging malignancies.

## Graphical Abstract

**Graphical Abstract:**
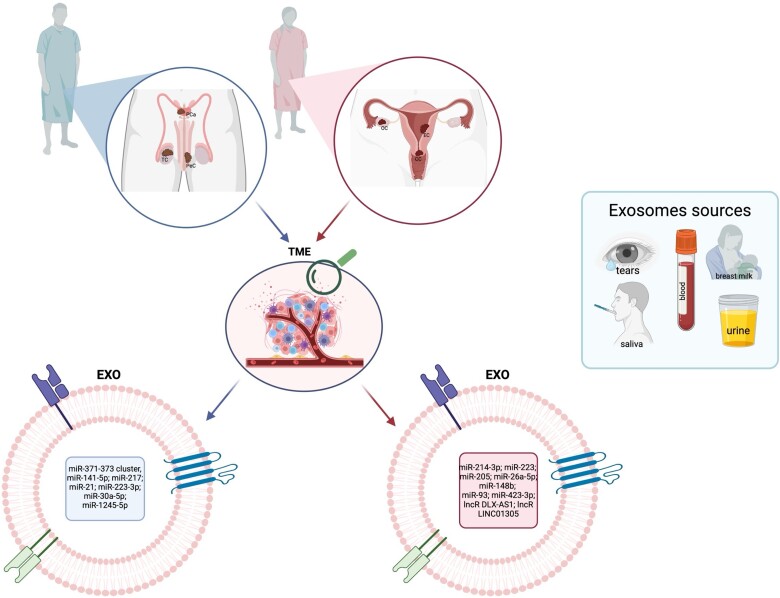

## Introduction

Cancers represent a major global health challenge that consistently ranks as a leading cause of death and disease worldwide [1]. These pathophysiological conditions emerge from the abnormal differentiation and proliferation of cells, primarily driven by genetic mutations. Such cells exhibit a dysregulated cell cycle, resist programmed cell death (apoptosis), and are characterized by uncontrolled growth and the capacity to metastasize [2]. A tumor is a vivid example of gender as an indicator of the pathogenesis, prognosis, and even diagnosis of many diseases [3]. Factors that expose the body to cancer include hormonal changes, age, obesity, genetic defects, inflammation, ultraviolet exposure, viral and bacterial infections, well-being, and general sanitary conditions, as well as the availability of health care [4, 5]. Other significant risk factors include smoking, alcohol consumption, environmental pollution, and a lack of physical activity [6–8]. For non-reproductive cancers, the prevalence in men is twice as high as in women. Some cancers, such as bladder cancer, appear up to four times more often in men than in women [9].

**Figure 1:**
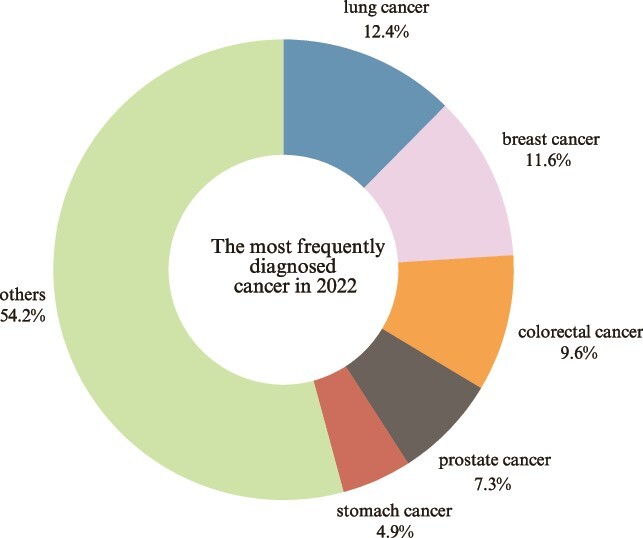
The frequency of most common cancers in 2022. Source: GLOBOCAN 2022 [1, 10]. *Created with Canva.*

As a major public health concern of the 21st century, cancer statistics from 2022 identify lung cancer as the most prevalent, representing 12.4% of all diagnosed cases, followed by breast cancer at 11.6%, colorectal cancer at 9.6%, prostate cancer (PCa) at 7.3%, and stomach cancer at 4.9% (Figure 1) [1]. Meanwhile, lung cancer is characterized by the highest mortality rate (18.7%), followed by colorectal (9.3%), liver (7.8%), breast (6.8%), stomach (6.8%), and pancreatic cancers (4.8%) [10].

**Figure 2:**
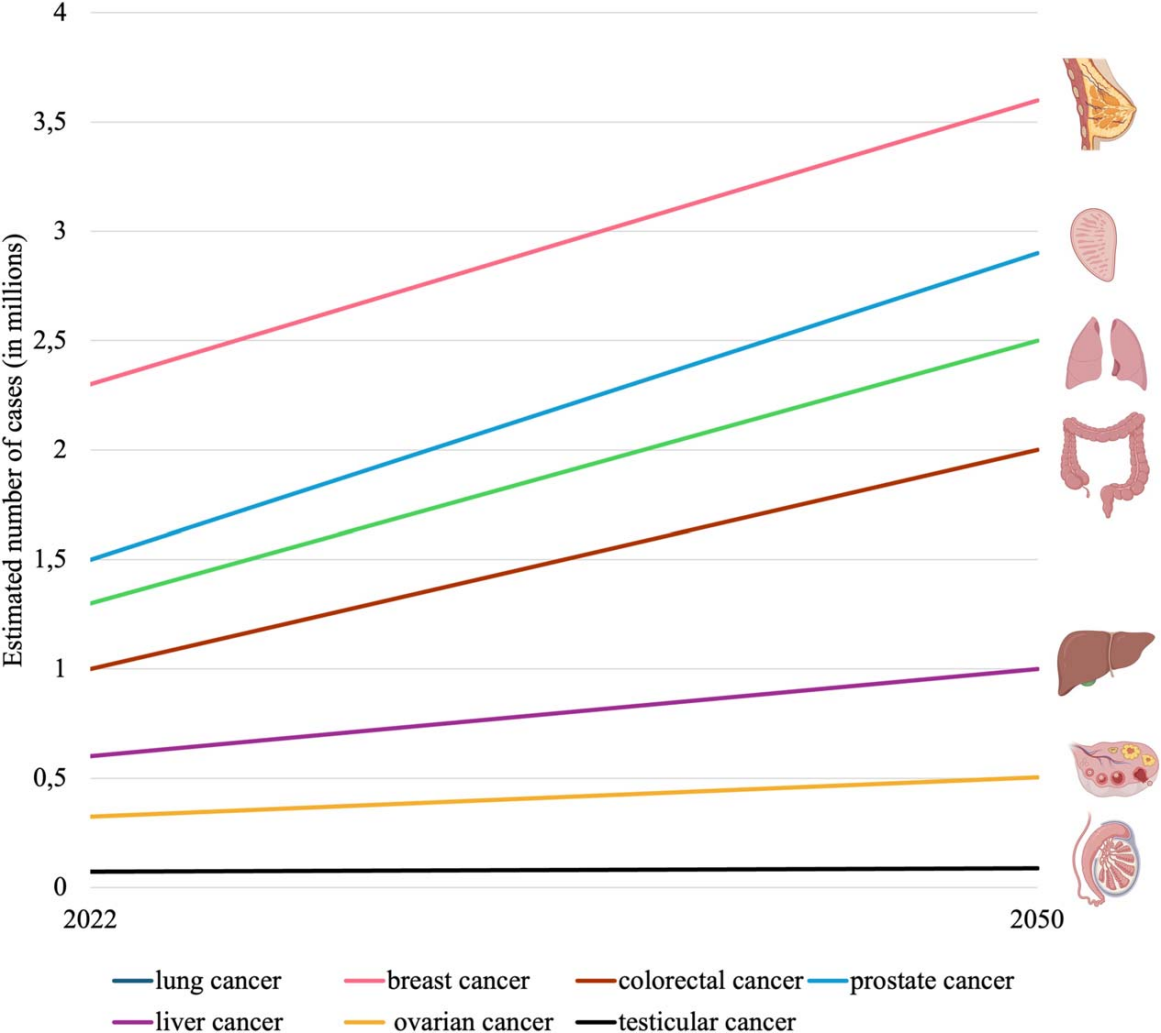
Estimated cancer cases (2022–2050). Source: GLOBOCAN 2022. [1, 10]. *Created with MS Excel and*  BioRender.com.

Year by year, there is an observed increase in cancer incidence rates [11]. It is estimated that by 2050, the incidence of various types of cancers will nearly double among both women and men [10]. For example, it is estimated that from 2022 to 2045, the number of cancer cases globally for both genders will increase, including lung cancer (up by about 65.0%), breast cancer (+36.9%), colorectal cancer (+60.0%), PCa (+65.0%), liver cancer (+66.0%), ovarian cancer (OC; +40.0%), and testicular cancer (TC; +7.0%) (Figure 2) [10]. It is projected that by 2040, over 27 million new cancer cases will be diagnosed globally, presenting a substantial public health challenge as the population ages [6, 8].

Moreover, the prevalence of tumors, including those affecting the genitourinary system, escalates with age [12]. With life expectancy on the rise, the impact of cancer spans all ages, making aging a primary risk factor for cancerous diseases [13, 14]. By 2050, it is estimated that over 20% of the global population will be aged 60 or older, posing economic, health, financial, and social threats [8, 15]. The relationship between cellular aging and the development of cancerous lesions, as well as the molecular changes that occur, are crucial areas of focus for cancer research and monitoring, leading to potential alterations in entire cellular systems [15].

Molecular research is currently focused on developing methods for the precise elimination of cancerous structures. Accurate knowledge of cellular relationships at multiple levels can be crucial [16–18]. Preclinical animal studies primarily use young organisms, i.e., relatively fresh cells, which may constitute an obstacle to obtaining objective data that can be translated into clinical cases [15]. Another important problem in cancer treatment is the knowledge of people participating in diagnostic procedures. Until recently, information came mainly from research on male bodies and male cell lines. Until 1993, the female sex was excluded from clinical trials, and it was assumed that male and female cells were biologically identical. This resulted in the need to withdraw a large number of drugs already in the first decade of the 21st century, as their adverse effect on the female sex was demonstrated [9, 19]. For the proper development of medicine and the success of clinically developed therapies, it seems necessary to characterize studies in terms of gender, due to differences in epidemiology, mortality, survival, pathophysiology, symptoms, response to treatment, and psychological effects [20, 21]. It is also absolutely necessary to know intracellular and intercellular relationships. The processes of aging, secretion, absorption, and communication at the molecular level have an indisputable impact on the development of most diseases. The formation and movement of certain structures, such as extracellular vesicles, is now such valuable knowledge that scientists devote entire research to them. Seeing their enormous potential, researchers emphasize the need to expand our knowledge of these particles [22–25].

Extracellular vesicles (EVs) have been the subject of extensive research over the past two decades and continue to be a significant area of interest for many researchers [26–28]. It has been shown that EVs are mediators during pathological conditions including cardiovascular diseases [29], neurodegenerative diseases such as Alzheimer disease [30], retinal degeneration [31], endometriosis [32], and uterine inflammation [33], as well as prostate dysfunctions [34]. Additionally, these vesicles contribute to dysfunctions in the reproductive systems of both genders and are linked to reproductive cancers [35].

The aim of this review was to discuss the importance of exosomes, in the carcinogenesis process and cancer progression in the female, as well as male, reproductive system.

## Extracellular vesicles

Extracellular vesicles are small lipid-bilayer enclosed particles released from cells into the extracellular matrix (ECM) [36]. They can be found in body fluids, such as blood, follicular fluid, semen, breast milk, saliva, tears, and urine [27, 37, 38]. EVs contribute to cell-to-cell communication; they act like messengers to the target cell [39]. They also contribute to the maintenance of homeostasis [29]. Characterized by their diverse cargo, EVs transport nucleic acids such as DNA and RNA, including miRNA, and messenger RNA (mRNA), as well as proteins, for example signaling proteins, enzymes, transcription factors, and also lipids [26]. This group includes distinct subtypes—exosomes (EXOs), micro-vesicles (MVs), and apoptotic bodies (ABs; *also referred to as apoptotic vesicles*) [40], which differ from each other in size, biogenesis mechanism, cargo, or even function [39]. EXOs are nano-sized vesicles with a restricted size of 30–150 nm, MVs have a diameter between 100 and 1000 nm, and ABs have a diameter between 1 and 5 μm [41, 42]. These differences are detailed in Table 1.

**Table 1:** Differences between subtypes of EVs

	Exosomes	Micro-vesicles	Apoptotic bodies	References
Origin	Exocytosis from multivesicular body (MVB)	Plasma membrane	Apoptotic cell	[43, 44]
Size	30–150 nm	100–1000 nm	1–5 μm	[41, 42]
Cargo	Nucleic acids, proteins, lipids, enzymes, metabolites	Cytosolic contents—lipids, nucleic acids, metabolites	Different fragments of organelles, chromatin, histones, degraded proteins	[43, 45, 46]
Role/Function	Cell-to-cell communication, transportation functions, biomarkers of diseases, modulation of immunological response, tissue regeneration, cancer progression, neurodegenerative diseases	Modulation of immunological response, blood coagulation, angiogenesis, cancer progression, interaction between distant or neighboring cell	Generated during apoptosis, cleaned by phagocytotic cells	[40, 44, 46, 47]
Markers	Tetraspanins (CD63, CD81, CD82, CD9), heat shock proteins (HSP60, HSP70, HSP90)	Integrins, selectins, CD40, Annexin A1	Annexin V, phosphatidylserine	[43, 44, 48]

**Figure 3:**
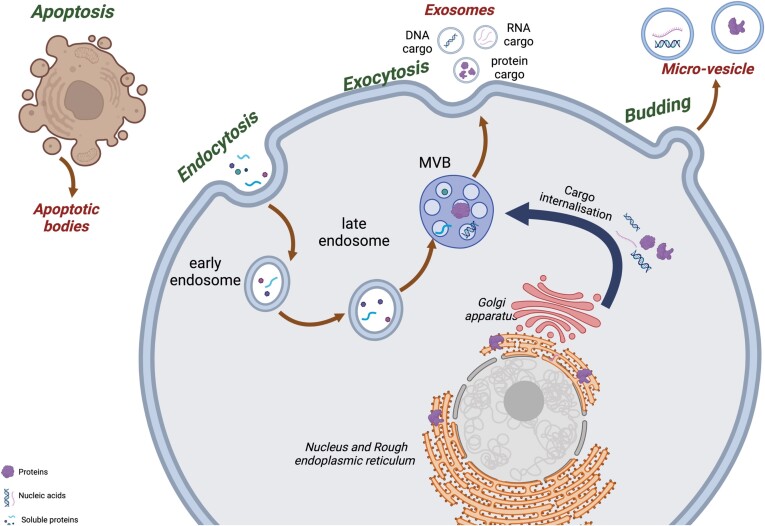
Biogenesis of EVs. *Created with*  BioRender.com.

Each subtype of EVs has a different mechanism of biogenesis (Figure 3). Exosomes originate from the fusion of the multivesicular body (MVB) with the membrane during exocytosis. Micro-vesicles are formed from cell membranes through budding, whereas apoptotic bodies are released during apoptosis [43, 49].

### Exosomes

**Figure 4:**
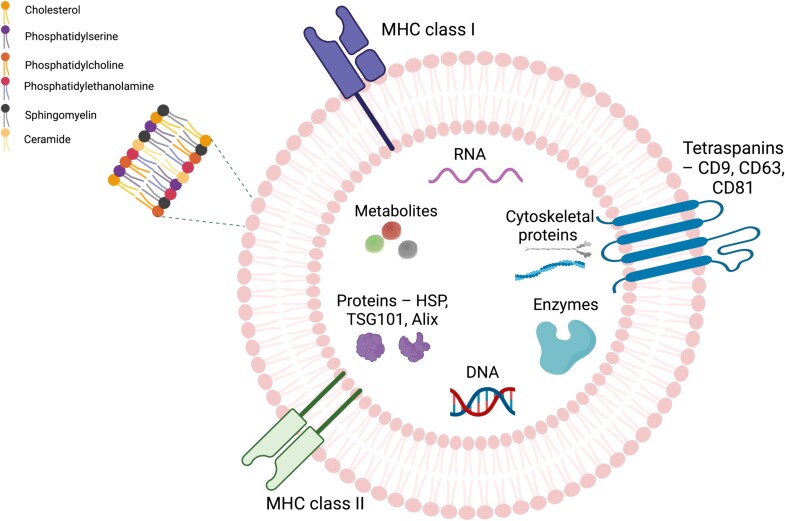
Structure and composition of exosomes. *Created with*  BioRender.com.

Exosomes are nanoscale vesicles, typically ranging from 30 to 150 nm in diameter. They are formed through a process that begins with endocytosis of the plasma membrane, leading to the development of early endosomes. These early endosomes eventually mature into MVBs [50]. Exosomes are derived from cellular membranes and thus contain a rich mixture of lipids such as phosphatidylserine (PS), phosphatidylcholines (PCs), phosphatidylethanolamine (PE), ceramide, sphingomyelin, and cholesterol **(**Figure 4**)** [51, 52]. The lipid bilayer of these vesicles serves as a protective barrier, shielding the cargo from enzymatic degradation during their transit from donor to recipient cells [53]. Moreover, their surface contains extracellular transmembrane proteins, such as Major Histocompatibility Complex (MHC) classes I and II, integrins, and tetraspanins (CD9, CD63, CD81). Exosomes also contain cytoskeletal proteins (e.g., actin, tubulin, myosin), enzymes (proteases), intracellular proteins, including integrins, and acetylcholonesterases, as well as MVB formation proteins like Alix and TSG101 [51, 52, 54, 55]. These proteins are recognized as markers for EVs enabling their identification [52]. Additionally, tetraspanins facilitate cell penetration and fusion, cytoskeletal proteins are involved in exosome release, and exosomes are enriched with heat shock proteins (HSPs), predominantly HSP70 and HSP90, which play a crucial role in stress response [51]. EXOs contain nucleic acids—DNA (single-stranded DNA, double-stranded DNA, mitochondrial DNA), and RNA, inducing messenger RNA (mRNA), microRNA (miRNA), small nuclear RNAs (snRNAs), small nucleolar RNAs (snoRNAs), transfer RNAs (tRNAs), ribosomal RNAs (rRNAs), and long non-coding RNAs (lncRNAs) [56].

The release of extracellular vesicles depends on specific conditions and can differ in both physiological and pathological states, including during diseases and the process of cancer development [26]. Therefore, they are potential biomarkers of a broad spectrum of diseases and conditions [49]. EXOs are secreted by various types of cells, such as endothelial cells and immune cells (lymphocytes, macrophages, dendritic cells [DCs], natural killer [NK] cells, fibroblasts, and epithelial cells [48]). EVs are also utilized for drug delivery purposes, including gene therapy applications. Exosomes are noted for their excellent biocompatibility, minimal immunogenicity, the capability to cross the blood–brain barrier, and the precision of direct drug delivery to the targeted therapy site [52].

Exosomes can activate a range of bioactivities in recipient cells through the binding of ligands on their membranes. This can occur through internalization, membrane fusion, or receptor–ligand binding. Internalization takes place via endocytic pathways, and fusion of the exosome with the plasma membrane occurs with the aid of Rab and Soluble NSF attachment protein receptors (SNAREs) proteins, which are necessary for this process. On the other hand, receptor binding on the cell surface occurs through ligands on the exosome surface, which carry MHC–peptide complexes and tumor necrosis factor (TNF), participating in the transmission of the immune response signal [57]. Receptor binding occurs in the case of exosomes released from DCs, which contain TNF and TRAIL (TNF-related apoptosis-inducing ligand). They bind to receptors on recipient cells and lead to the activation of T cells, triggering an immune response and inducing apoptosis [58]. Exosomes absorbed through processes such as endocytosis, phagocytosis, or fusion with the plasma membrane of the recipient cell allow for the direct release of their cargo into the cell and bypass lysosomal degradation [54]. There is also the possibility for exosome cargoes to diffuse into the cytoplasm, which may facilitate RNA transport [54]. The cytoplasmic reticulum is a deposit site for mRNA and miRNA [59].

Exosomes significantly contribute to intercellular communication and are produced by both healthy and defective cells [60]. Research has demonstrated that exosomes are crucial in immunological regulation, tissue repair, angiogenesis, and cellular signaling, and they also play roles in reproductive processes [61, 62]. In our previous paper, we extensively reviewed the involvement of exosomes in the reproductive systems of both genders, covering aspects such as oogenesis, embryo implantation, fertilization process, sperm maturation, capacitation, and acrosomal reaction [63].

### Exosomes in cancer growth

Besides participating in physiological functions, exosomes also play a crucial role in cancer development [64]. The vesicles released from tumors are referred to as tumor-derived exosomes (TEXs) [65]. Tumor-derived exosomes are characterized by generating immunosuppression, uncontrolled cell growth, angiogenesis, and metastasis. Moreover, these particles can be used as cancer biomarkers [66]. The cargoes carried by exosomes released from healthy cells and those from the tumor microenvironment differ [65]. Moreover, cancer cells secrete more exosomes than other cells [67].

The release of exosomes and the nature of their cargo vary depending on the cell type, growth status, or receptor stimulation [68]. Research has shown that exosomes play a critical role in mediating communication between cancer cells and the surrounding stromal cells by delivering non-coding RNAs (ncRNAs) [53]. Despite the fact that ncRNAs do not encode proteins, these molecules participate in various physiological and pathological processes, including the life activities of cells and the development of diseases [12]. They are pivotal in evaluating the efficacy of cancer treatments, offering a method for non-invasive early detection and diagnosis of cancer through their role as biomarkers [69]. Additionally, they are key in developing therapeutic strategies to halt cancer progression and support the immune system’s strength [70].

These ncRNAs are implicated in the onset and development of various cancers, including those affecting the reproductive organs [12, 71]. The ncRNA group includes microRNAs (miRNAs), lncRNAs, and circular RNAs (circRNAs), each playing distinct regulatory roles [72]. MiRNAs are involved in regulating gene expression at the post-translational level and cell proliferation and influence genes and signaling pathways [73]. In turn, lncRNAs contribute to the establishment of disease states, and circRNAs, which are cell-specific, often show altered expression patterns during the process of carcinogenesis, potentially affecting the spread of cancer. Exosomes, enriched with ncRNA, can traverse through bodily fluids from donor cells, such as cancer cells, to distant recipient cells, significantly impacting tumor growth, proliferation, progression, and metastasis [74–76]. This exosomal pathway helps cancer metastases evade the immune system [75]. Numerous studies have indicated that exosomal ncRNA can be employed as a biomarker for various cancers, including breast cancer [77], lung cancer [53], gastric cancer [78], and colorectal cancer [79]. There is increasing research proving the role of exosomes and ncRNA, especially miRNA, in genitourinary tumors [12, 80].

The tumor microenvironment (TME) provides a unique setting for communication between cancerous and non-cancerous cells through messengers like proteins, lipids, and ncRNAs, which are transported within exosomes as their cargo [81]. Moreover, the TME is involved in cancer growth [12]. The TME comprises cancer cells, fibroblasts, immune cells, and products of secretion like cytokines and chemokines, as well as metabolites (e.g., lactic acid) and blood vessels [50, 82, 83]. This environment is characterized by low oxygen levels and high acidosis due to lactate content [84]. The TME also includes immune cells, notably tumor-associated macrophages (TAMs), which are the most abundant [85]. Macrophages are categorized into two types—M1 and M2. M1 macrophages, which secrete pro-inflammatory cytokines like lipopolysaccharide (LPS) and interferon-gamma (IFN-γ), combat cancer, whereas M2 macrophages produce cytokines that support anti-inflammatory responses and aid in tumor progression [86, 87]. Moreover, M2 macrophages play a role in angiogenesis, tissue repair, and tumor progression [86]. Cytokines like IL-4 and IL13 promote M2 macrophage polarization within the TME [88]. It has been proven that macrophages within the TME can spontaneously transform from the M1 to M2 phenotype [86]. Exosomes have been shown to influence the polarization of these macrophages and promote tumor growth [86, 87]. Tumor-associated macrophages are known to promote tumorigenesis and are associated with poor prognosis [85]. Cancer cells may encourage TAMs to adopt an M2 phenotype, while TAMs can infiltrate tumors utilizing exosomes laden with miRNAs that are essential for cancer progression [85].

The functions of exosomes vary depending on the tissue or cell from which the vesicles originate [89]. It has been proven that cells that are linked to the cancers secrete more extracellular vesicles than other, non-cancer, cells. This occurs to meets the need for nutrient supply and transfer of information. Cancer patients have almost twice the content of EXOs in their blood compared to healthy individuals [90]. Exosomes and their bioactive cargo can be involved in the regulation of intercellular communication between tumor cells and the TME and participate in angiogenesis [12]. Moreover, exosomes released by tumors are important in immune modulation because of their suppressive cargo [91]. Exosomes contribute to promoting tumor growth, angiogenesis, and preparation of pre-metastasis niches.

#### Promoting tumor growth

Exosomes released by cells retain the properties of the donor cells and can thus modulate the recipient cells, as is the case in cancers [92]. Cancer EVs have been shown to carry oncogenic proteins, RNA, and DNA [93]. Additionally, they can carry bioactive tumor molecules that, when taken up by recipient cells in the TME, can contribute to immune tolerance or unresponsiveness. In contrast, EVs derived from immune cells can inhibit tumor growth and proliferation [93]. It is also possible to activate signaling pathways that promote cell proliferation, such as the PI3K/Akt and mitogen-activated protein kinase (MAPK) pathways [94]. Activation of the phosphoinositide 3-kinase (PI3K/AKT) pathway occurs mainly in cancers and translates into the promotion of tumor cell growth. In turn, the MAPK pathway has been shown to promote tumor cell migration [94]. Research indicates that the disruption of signaling pathways such as Ras/Raf/MEK/ERK, p53, mammalian Target of Rapamycin (mTOR), and STAT3 in cancer may impact exosome release and subsequent proliferation. MEK and ERK kinases, frequently altered by the Ras and Raf kinases through small G proteins, play significant roles in cancer proliferation, invasion, and metastatic spread [95]. The mTOR

signal pathway is important in maintaining cell proliferation, growth, and survival [96]. Similarly, the STAT3 pathway contributes to cancer growth, survival, metastasis, and angiogenesis during tumor development [97]. Additionally, mutations in the tumor suppressor protein p53, present within exosomes, are linked to increased metastatic potential and resistance to cancer therapies [98].

#### Angiogenesis

Exosomes can promote the formation of new blood vessels (angiogenesis) by carrying pro-angiogenic factors such as VEGF (vascular endothelial growth factor), TNF-α (tumor necrosis factor alpha), and miRNAs that regulate angiogenic pathways. This provides increased blood supply to the tumor, facilitating its growth and expansion [99, 100]. Exosomes released by fibroblasts enhance tumor activity, linked to increased cancer cell invasiveness and the facilitation of metastasis [12]. Furthermore, hypoxia in the tumor environment induces the expression of hypoxia-inducible factors (HIFs), which subsequently modulate the process of blood vessel formation [101]. Pro-angiogenic factors act on endothelial cells, which promotes proliferation. It has been shown that tumor-derived exosomes carry pro-angiogenic factors, and, when taken up by endothelial cells, they can stimulate the process of blood vessel formation, which may be important for tumor development [99].

#### Epithelial–mesenchymal transition

Exosomes contribute to cancer metastasis by participating in the regulation of epithelial–mesenchymal transition (EMT) and remodeling of the ECM, a component of the tumor environment [93, 102]. Extracellular matrix remodeling is a process in which epithelial cells lose their cellular polarity and adhesive properties and gain migratory and invasive properties. During this process, tumor epithelial cells, under the influence of cancer-associated fibroblasts (CAFs) in the tumor matrix, transform and acquire mesenchymal characteristics [93]. Cancer-associated fibroblasts have been shown to promote cancer progression and indicate drug resistance properties by reshaping the ECM into a physical barrier that impedes drug penetration [102]. Interactions between CAF, tumor, and mesenchymal cells take place with the participation of exosomes, and the stimulants are transforming growth factor beta (TGF-β), fibroblast growth factor (FGF), platelet-derived growth factor (PDGF), interleukin-1 (IL-1), and interleukin-6 (IL-6) [103]. It has also been shown that a group of Wnt glycoproteins, which are important in the development of neoplastic diseases, is important in the context of communication between stromal and epithelial cells [104]. The transport of these hydrophobic molecules is facilitated by exosomes [104]. Wnt then activates the Wnt/β-catenin pathway, which allows the preservation of tumor stemness [104].

#### Preparation of pre-metastatic niches

Tumor-derived exosomes can travel to distant organs and modify the local environment to create a pre-metastatic niche [105]. Furthermore, in the bone marrow, exosomes can contribute to cell–cell interactions due to their regular migration through the circulatory system and promote metastasis by altering the ECM and modulating local immune cells, making the distant site more conducive to cancer cell colonization [105, 106].

#### Integrin-dependent organotropism

Cancer metastasis is not random but depends on a series of controlling cells that have the ability to colonize and metastasize [107]. Exosomes have specific integrins in their structure, which define their target site in the body and can contribute to exosome biogenesis [108]. Exosomal integrins are involved in the process of carcinogenesis, particularly in preparing metastatic niches or modulating angiogenesis [108]. For example, exosomal integrins α6β4 and α6β1 are associated with lung metastasis, while those with αvβ5 integrins tend to direct metastases to specific organs, including the liver [107]. This specificity helps to direct metastases to specific organs. Integrins α6β1 in exosomes derived from pancreatic tumors can result in lung metastasis [109]. Integrin β1 is associated with PCa. In addition, α2β1 has been shown to reduce proliferation and increase invasion of this type of cancer [110]. Also, in cervical cancer (CC), increased expression of integrin β1 has been reported [111].

Another pathway of exosome’s action during tumor growth is their impact on DCs, which are important in immune responses against cancer and in maintaining immune tolerance. It has been demonstrated that TEX inhibits DC differentiation from bone marrow progenitors, thus weakening anti-tumor reactions. Moreover, components of the tumor’s exosomes, such as IL-6, can participate in the reduction of maturation of T cells. Tumor-derived exosomes can also disturb TNF-α and IL-12 production, which is crucial for T-cell activation [86].

**Figure 5:**
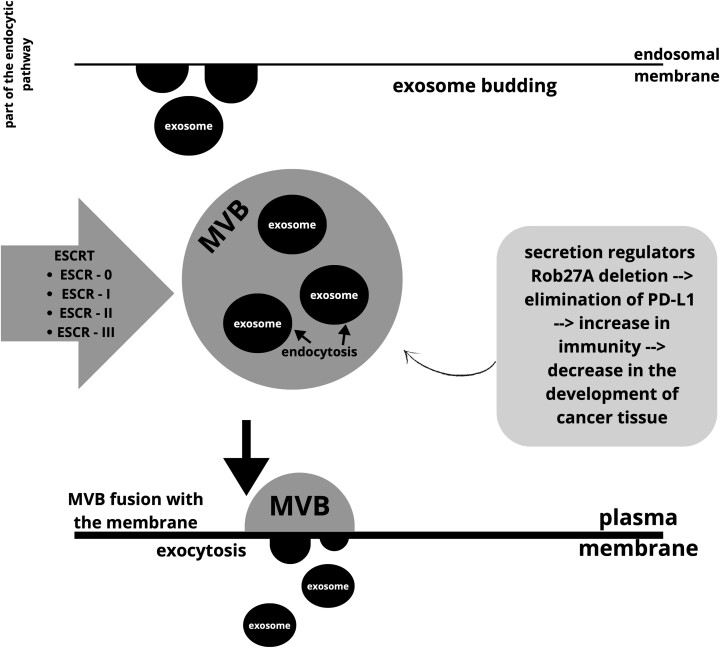
The process of exosome formation. *Created with*  Canva.com.

According to data from the exosome database, there are currently almost 10 000 proteins, over 3400 mRNA structures, over 2800 miRNAs, and over 1100 lipids [112]. The process of exosome formation (Figure 5) involves budding into the endosomal membrane, leading to the accumulation of vesicles in larger multivesicular bodies. For this reason, exosomes are classified as part of the endocytic pathway. Transmembrane-type proteins, such as exosomes, must initially be endocytosed and transferred to the early stages of endosomes. After the MVB fuses with the plasma membrane, the particle in the form of an exosome is released outside the cell through exocytosis. The entire process is influenced by factors influencing the formation of MVB structures but also the maturation of the endosomes themselves. One of the best-known factors of this type is the endosomal sorting complex required for transport (ESCRT). The structure of ESCRT is composed of several complexes: ESRT-0, ESRT-I, ESRT-II, and ESRT-III. The mechanism is designed to sort ubiquitinated cargoes that will be used for the lysosomal degradation process. Regulators of exosome secretion may affect MVB fusion with the membrane. Additionally, it has been shown that deletion of one of such regulators—Rab27A, leading to the elimination of exosomal programmed death-ligand 1 (PD-L1), can block the development of cancer tissue by stimulating anti-tumor immunity [26, 59, 113, 114].

### Tumor progression modulators associated with exosomes

Exosomes transport a variety of cargo, including bioactive molecules such as proteins, lipids, RNA, and DNA that can influence various aspects of tumor biology, such as tumor growth, metastasis, immune evasion, and drug resistance [83, 115]. Modulators associated with cancer progression and exosomes may include a wide group of factors [116].

#### MicroRNA

Extracellular vesicles transporting miRNAs are associated with drug resistance. Administering it to drug-sensitive cancer cells complicates the treatment because it causes them to become desensitized to the administered medicinal substances. It is noted that the transfer of MDR-1/P-glycoprotein by this type of exosomes resulted in increased resistance to the administered drug. At the same time, exosomes have the ability to bind to drugs, which inhibits the reaction to the administered substance [116]. In addition, exosomal miRNAs are associated with the preparation of niches and components of the TME. They also participate in cell–cell communication and can be markers of cancer diseases [117].

#### Long non-coding RNA

Long non-coding RNAs play an important role in the process of oncogenesis. Long non-coding RNAs are involved in the regulation of many biological processes, such as apoptosis and proliferation, cell cycle, and cell proliferation [118]. At the same time, however, they may be expressed incorrectly during tumor formation. As a result, some lncRNAs act as tumor promoters or act as tumor suppressors. The functional role of lncRNAs is not fully understood, especially their involvement in carcinogenic processes [119].

#### DNA

Oncogenes such as C-myc exhibit specific mechanisms of cellular deregulation: patterns of amplification, mutation, and translocation. These reactions and changes in the genetic material characterize malignant tumors. The C-myc-related translocation process is occasionally combined with a second process—amplification or mutations. This type of deregulation caused by C-myc may influence clinical changes in cancer cells. As a result, it is necessary to modify therapeutic strategies [120].

#### Proteins

Exosomes released from cancer cells contain TGF-β, PD-L1, and epidermal growth factor receptor (EGFR) [121]. The concentration of TGF-β3 protein in EVs is a strong predictor of response to chemoradiotherapy. Exosomes isolated from the plasma of cancer patients contained, among others, significant amounts of TGF-β, OX40 (CD134), OX40L (CD134L), and HSP70. Particles of this type induce the process of apoptosis. As a result of this reaction, they can regulate the immune response. In the case of cancer patients, this leads to fueling the carcinogenesis process. Anti-PGE2 and TGF-β antibodies do not allow the induction of Myeloid-Derived Suppressor Cells (MDSCs) and, as a result, weaken the capacity of MDSCs. Knowledge of these molecular relationships may prove helpful in developing targeted tumor treatment strategies [122].

#### Lipids

Sphingolipids are involved in apoptosis and the development of cancer cells. This happens because these particles control the transmission of signals by cancer cells [123]. In tumor tissues, the recruitment of non-malignant cells, e.g., immunosuppressive cells, is induced by lipid mediators such as prostaglandin E2 (PGE2). Prostaglandins themselves are also synthesized in this type of cells. This may lead to the development of an immunosuppressive TME [124].

#### Cytokines

Cytokines such as IL-6 can induce mRNA expression in cancer cells. Wei et al. [125], in their study on mice, showed that IL-6 promoted the growth and development of CC due to angiogenesis. It has also been shown that colorectal cancer cells are able to produce IL-6 on their own. Stimulation of these cells with another type of cytokine (IL-17) also causes these cells to produce IL-6, which further stimulates angiogenesis. This process is directly related to the development of cancer tissue. Some CXC chemokines also show this type of activity, but, additionally, they are based on the ELR (Glu-Leu-Arg) motif. The presence of this motif in the particle promotes angiogenesis, while its absence inhibits it. Thus, the chemokines CXCL1, CXCL6, CXCL8, and CXCLR5 are ELR-positive and promote angiogenesis, while CXCL4, CXCL10, and CXCL14 are ELR-negative and thus inhibit angiogenesis. The chemokine CXCL12 is an exceptional case in this situation because it is characterized by an ELR-negative model and nevertheless induces the angiogenesis process [126].

#### Integrins

Cell adhesion receptors are integrins; their function depends on biochemical reactions inside the cell. As signalers, integrins bind extracellular ligands. In the case of signals coming from the extracellular environment, integrins collect information and transmit it inside. In healthy tissue, these particles act as checkpoints and monitor the effect on proliferation. In the case of cancer tissue, this effect changes. A change in the internal environment of the cell may cause disruption of metabolic processes, including oxygen. Lack of sufficient amounts of these particles increases the production of certain integrins. This may be related primarily to cancer metastases and a reduction in the patient’s prognosis. Changes in the processes related to signaling between the internal and external environment of the cell may cause changes in integrin, e.g., the transformation of α3β1-integrin to α6β4, which indirectly leads to the differentiation of cancer cells. Also, changes in αvβ3 surface integrins may increase the migration of cancer cells [127].

#### Signal transduction molecules

Signal transduction molecules, in the case of their mutations or overactivity, forms of EGFR are noticed in cancer tissues. Biochemical mechanisms involving EGFR may promote the development of cancer tissue in the event of such an abnormality. In cancer treatment, some substances specifically block EGFR reaction pathways. However, some patients do not respond to therapies based on anti-EGFR drugs. Some patients also become tolerant to these types of substances to the point that they stop reacting to them [128].

#### Mutation in Mitochondrial DNA (mtDNA)

Mutations in mtDNA affect cancerous tissues and often result in a deterioration of the patient’s health. Mitochondria play a key role in the biophysical state of the cells examined. At the same time, mtDNA may be a potential target for anticancer therapies [129].

#### Matrix metalloproteinases

Matrix metalloproteinases (MMPs) MMP-2 and MMP-9 are involved in the degradation of the ECM. This leads to simplified invasion of cancer cells and, consequently, tumor metastasis. Effects on these metalloproteinases can block tumor growth and cell invasion. Inhibiting the transformation of these particles seems to be a potential therapeutic strategy [130].

## The role of exosomes in male reproductive cancers

**Figure 6:**
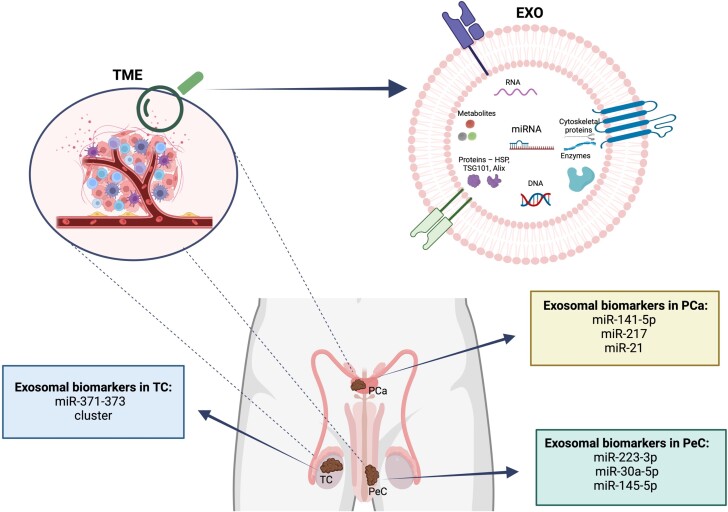
Male reproductive cancers and their exosomal potential biomarkers. *Created with*  BioRender.com. Legend: PCa—prostate cancer, PeC—penile cancer, TC—testicular cancer, TME—tumor microenvironment, EXO—exosomes.

Semen is abundant with exosomes that enhance sperm motility [131] and capacitation [132] and also help inhibit premature acrosome reactions [133, 134]. Prostasomes are the exosomes derived from the prostate, while exosomes from epididymal fluid are referred to as epididymosomes [63]. Exosomes, particularly prostasomes, play a protective role in the female reproductive system by modulating sperm activities. These prostasomes enhance sperm motility and capacitation, manage acrosomal reactions, and prevent premature capacitation by delivering cholesterol and sphingomyelin. Additionally, epididymosomes from the epididymis are crucial for increasing sperm’s fertilization capabilities, protecting them from oxidative stress, and controlling their movement [63]. They also can affect spermatogenesis due to their ability to transfer the molecules that can improve communication within the testes structures such as Sertoli cells and Leydig cells [61, 135]. These vesicles can indicate dysfunctions and conditions around male reproductive system [134]. In 2022, the most abundant cancer among men was lung cancer; the second was PCa [10]. Cancers within the male reproductive tract (Figure 6) can lead to infertility, overall health issues, and reduced semen quality in men [136].

### Prostate cancer

Prostate cancer ranks as the second most common cancer in men [137] and stands fifth in global cancer mortality rates [138]. Risk factors include age, obesity, smoking, and genetic predispositions [139]. This is the most commonly detected cancer in men over the age of 50 [140]. Prostate-specific antigen testing, currently used for diagnosis, shows limited specificity, leading to potential false positives [141]. The development of liquid biopsy techniques, which utilize the isolation of EVs as potential biomarkers, shows promise for more accurate diagnostics [142].

To better identify markers for disease, researchers are studying exosomal miRNAs. In their study, Li et al. [143] collected blood from 31 PCa patients and 19 healthy men, isolating exosomes to analyze for miRNA-125a-5p and miR-141-5p. They found elevated levels of miRNA-141-5p in patients with PCa compared to healthy individuals, whereas miRNA-125a-5p levels were significantly lower in patients than in healthy controls. Moreover, the ratio of miR-125a-5p to miR-141-5p was higher in men with PCa than in those from the control group [143]. It has also been shown that exosomal miR-141-3p from prostate cancer modulates the cancer microenvironment and contributes to the development of PCa bone metastasis [144]. The researchers suggest that high levels of exosomal miR-141-3p and low levels of miR-125a-5p could serve as biomarkers for this type of tumor [143]. In the study conducted by Xu et al. [145], differences in miRNA-141 expression from EVs collected from urine and blood serum were analyzed in benign prostatic hyperplasia (BPH) and PCa. The authors did not detect significant differences [145].

In a study conducted by Zhou et al. [146], exosomes isolated from the blood of 10 prostate cancer patients and 10 healthy individuals revealed that levels of miR-217 were elevated in the patient group compared to the healthy group. The research indicated that higher levels of miR-217 contribute to the proliferation and invasion of cells [146].

Reports indicate that miR-21 and miR-375 show varied expressions in cases of PCa [147]. In studies conducted by Joković et al. [148], blood was collected from 35 prostate cancer patients and 34 men with BPH, and exosomes from the patients’ plasma were analyzed for the expression of miR-21 and miR-375 in aggressive and non-aggressive forms of the tumor. Nearly three times higher expression levels of exosomal miR-21 were found in patients with aggressive forms of the tumor compared to less aggressive PCa. No differences were observed for miR-375. The researchers suggest the potential use of miR-21 as a prognostic marker in PCa [148]. It has been proven that miR-21 can participate in proliferation and tumor cell apoptosis by signaling pathway PTEN/PI3K/AKT, which can be a key target for chemotherapy [149]. Overexpression of miR-21 may be associated with EMT and lead to tumor malignancy. miR-21 can be successfully used as a biomarker of this type of cancer, but its molecular regulatory pathways need to be explored [150].

### Testicular cancer

According to 2022 data, TC ranked 27th in the incidence of cancers [10]. However, it is one of the most common malignant diseases among young men aged 20–40 [140, 151]. Ninety-five percent of TC diagnoses involve germ cell tumors (GCTs), while the other 5% comprise sex cord–stromal tumors that typically develop from Leydig or Sertoli cells [152, 153]. However, GCTs are the most commonly encountered type [152]. Furthermore, within GCTs, there are classifications of seminomas and non-seminomas [153].

The miR-371-373 cluster is considered a potential marker for testicular cancer, as it shows higher expression in patients with this condition [154]. In the research conducted by Alonso-Crisostomo et al. [80], the significance of exosomes in the context of testicular GCTs (TGCTs) and their TME was explored. This involved employing five distinct cell lines that represent various levels of TGCT malignancy, along with cultures of human testicular fibroblasts (symbolizing healthy tissue), human umbilical vein endothelial cells, and human macrophages to define the tumor’s environment. Exosomes were subsequently extracted. The investigation pinpointed crucial miRNAs, particularly those in the miR-371 ~ 373 and miR-302/367 clusters, which displayed significant elevation in TGCT-derived EVs compared to control samples [80]. These miRNAs appeared prevalently across different TGCT subtypes, highlighting their possible involvement in tumor dynamics and interactions with the TME. Experiments involving co-culturing and further analysis demonstrated that miRNAs transmitted through EVs from TGCT cells could notably modify miRNA profiles in TME cells such as fibroblasts and endothelial cells. These alterations were shown to impact critical cellular functions and activate pathways within the recipient cells, showcasing the significant effects of EV-mediated miRNA transport. Moreover, the overexpression system of the miRNA (miR-371a-OE) also induced angiogenesis in endothelial cells, suggesting that exosomes released from testicular cancer could facilitate tumor progression [80]. Studies [155] within this cluster have shown that the dynamics of expression in tumors were higher for miR-371a-3p compared to miR-372-3p and miR-373-3p.

### Penile cancer

Penile cancer (PeC) is not a commonly diagnosed tumor, ranking 30th in cancer prevalence in 2022 [10]. It predominantly affects men in developing regions [156]. The majority of these cancers are squamous cell carcinomas (SCCs), typically occurring on the glans or the foreskin [140]. Characterized by a gradual spread to the inguinal lymph node metastasis (LNM), this cancer often progresses to more extensive metastasis [157]. Metastatic presence in lymph nodes serves as a prognostic factor for patient outcomes [158]. Key risk factors include smoking, inadequate hygiene, and chronic inflammation, although most SCC cases are associated with the human papillomavirus (HPV) [156].

Research by Ayoubian et al. [159] aimed to identify miRNAs associated with the development of SCC and their connection to HPV infection. In this study, 27 cancer tissue samples and 18 normal tissues as controls were collected from patients for RNA extraction. Of the cancer samples analyzed, 12 were HPV-negative and 10 were HPV-positive. Significant differences in the expression of 876 miRNAs (*P* ≤ 0.01) were found in HPV-positive SCC tissues compared to HPV-negative SCC tissues. Furthermore, variations in the expression of 118 miRNAs (*P* ≤ 0.01) were observed when comparing SCCs with metastasis to those without metastasis. The lowest expression levels in metastatic cancer tissues compared to non-metastatic ones were noted for miR-137 and miR-328-3p. These results suggest that HPV infection may influence miRNA expression in PeC [159].

A research initiative examined the relationship between miRNA levels and LNMs using 50 formalin-fixed, paraffin-embedded SCC samples [160]. This study measured the expression of miR-223-3p and compared it to non-cancerous cells within the same samples. Significantly higher levels of miR-223-3p (*P* < 0.001) were found in primary tumor samples from patients with LNMs than in non-cancerous tissues. Additionally, miR-223-3p levels were notably higher in samples from metastatic lymph nodes than in the primary tumors of the same patients [160]. The authors suggest that miR-223-3p levels may be a marker for LNM [160]. Additionally, higher levels of elements at metastatic sites of cancer enable faster and non-invasive detection of subsequent changes in individual exposed to them, which is a potential new method of monitoring patient health [161].

In a study aimed at determining the role of miRNAs in the carcinogenesis and growth of penile cancer, Furuya et al. [162] collected tissue samples from 24 cancerous and 24 non-cancerous sources. During the research, 83 miRNA transcripts were profiled, and differential expression was found in 8 of them. Specifically, miR-31-5p exhibited increased levels, whereas miR-30a-5p, miR-432-5p, miR-487b-3p, and miR-145-5p showed decreased expression in cancerous tissues compared to non-cancerous ones. The study highlights that the expression disparities in miRNAs such as miR-30a-5p, miR-432-5p, miR-487b-3p, and miR-145-5p could help distinguish between cancerous and non-cancerous conditions. The authors suggest the potential use of these miRNAs as biomarkers for diagnosing and understanding the progression of PeC [162].

## The role of exosomes in female reproductive tract cancers

Exosomes play a pivotal role in the reproductive processes within the female reproductive tract. Originating from cells of this system, they are involved in regulating transcriptional activities, enhancing cellular growth and differentiation, and supporting oogenesis and oocyte maturation [63]. The follicular fluid contains numerous molecules and EXOs. These structures have been shown to play a significant role in the process of follicle regulation and maturation [118, 163]. Furthermore, these vesicles are crucial for embryo implantation and help sustain pregnancy through hormonal regulation. Due to their origin-specific nature, EXOs also function as potential biomarkers for detecting disorders such as polycystic ovary syndrome (PCOS) and various reproductive cancers [63].

**Figure 7:**
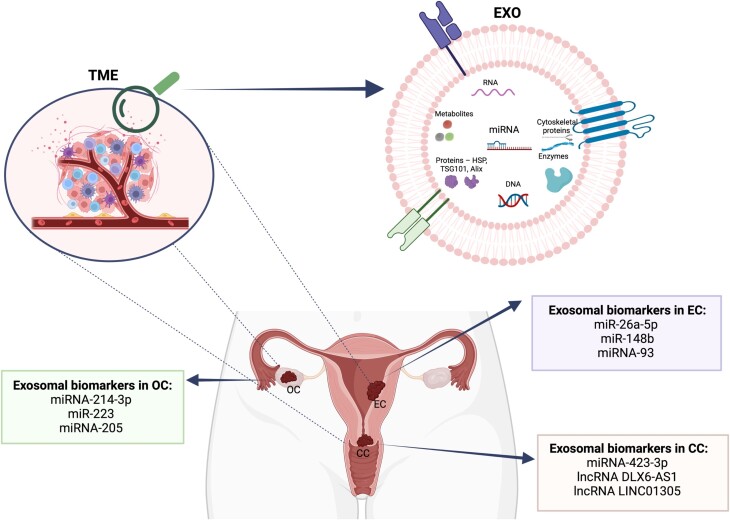
Gynecological cancers and their exosomal potential biomarkers. *Created with*  BioRender.com

In 2022, breast, lung, and colorectal cancers were the most common among women, with CC ranking as the fourth most prevalent [10]. Gynecological cancers pose a significant threat to women’s health, impacting reproductive organs like the uterus, ovaries, and cervix (Figure 7) [75, 164]. These cancers are highly lethal and present diagnostic challenges. While cancer metastasis is typically thought to occur via the lymphatic or circulatory systems, there is growing evidence that exosomes also play a role in this process [164]. Among gynecological malignancies, OC is the most lethal of all conditions in women; in the second place ranks CC [165].

### Endometrial cancer

Endometrial cancer (EC) is a very common malignancy of the female reproductive tract [166]. The risk factors of this type of condition include PCOS, infertility, estrogen exposure, and obesity and usually occur after menopause [167, 168]. The majority of EC cases occur in women between the ages of 65 and 75 [169]. The prognosis is poorer in cases with lymphatic metastasis [170]. Exosomes carrying miRNA contribute to the pathophysiology of EC by originating from tumor cells [169]. These oncogenic miRNA serves as a biomarker for EC [171].

Endometrial cancer arising in the lining of the uterus is divided into the more common endometrial EC (80% of patients) and the rarer non-endometrial EC (20% of patients). Exosomal miRNAs as biomarkers may play a key role not only in detecting the appearance of cancerous tissue but also the occurrence of metastases to other places in the body. This potential biomarker is also a valuable indicator in screening, diagnostic, and immune studies [169, 171, 172].

In research conducted by Wang et al. [170], it was found that the level of exosomal miR-26a-5p was lower in patients with EC, especially those with LNM human lymphatic endothelial cells (HLECs), compared to healthy individuals. The researchers also observed that transferring miR-26a-5p to HLECs could promote lymphangiogenesis in vitro*.* Furthermore, in a mouse model, the application of exosomal miR-26a-5p was shown to inhibit the proliferation of EC tumors and their metastasis to lymphatic vessels [170].

Research has shown that the development and migration of endometrial cancer may occur through CAFs, which produce exosomes carrying miRNAs [173]. In studies, 23 samples of EC were obtained from which CAFs were isolated, and, from a control group without cancer changes, normal fibroblasts (NFs) were isolated. An in vivo study was conducted on immunodeficient BALB/C mice to observe cancer progression. Throughout the study, the expression of miR-148b, a key regulator of EC progression, was assessed. It was found that miR-148b levels were lower in the culture of tumor-associated fibroblasts and EC compared to normal fibroblasts. Researchers argue that reduced levels of this miRNA in EC cells and the TME may indicate disease progression and a poor prognosis. In mice injected with NF, CAF, or CAF overexpressing miR-148b, after 4 weeks, CAFs significantly promoted the formation of EC metastases to the lungs, while overexpression of miR-148b limited the ability of CAFs to promote proliferation. Exosomes obtained from CAFs enhance cancer proliferation [173]. Li et al. [173] demonstrated that overexpression of miR-148b in endometrial tumor cells could result in inhibition of tumor progression.

In additional studies, the impact of exosomal miRNA-93 on EC was explored by Zheng et al. [174]. For this research, blood samples were collected from 100 patients with confirmed EC. These patients exhibited higher levels of exosomal miRNA-93 compared to healthy subjects, and, notably, higher levels were also observed in patients who smoked. This suggests that exosomal miRNA-93 levels could serve as prognostic indicators for the condition [174].

### Ovarian cancer

Ovarian cancer ranks as the eighth most common cause of cancer deaths among women globally [175]. It primarily manifests in epithelial and mesenchymal forms, with the mesenchymal type accounting for over 90% of OC cases [176]. Almost half of the high-grade ovarian tumors are associated with mutations in the *BRCA1* and *BRCA2* genes [176]. Exosomes released from OC cells carry crucial proteins such as EpCAM, CD24, and cancer antigen 125 (CA125), which are significant for the development of this cancer type. [177, 178].

A study conducted by Yang et al. [76] analyzed miRNA-214-3p levels in OC tissues and serum from 29 patients. They found higher expressions of miR-21-5p, miR-141-3p, miR-200a-3p, miR-200b-3p, miR-203-3p, miR-205-5p, and miR-214-3p in both low- and high-malignancy OC tissues compared to benign tumors, particularly noting the highest expression of miRNA-214-3p in high-grade epithelial OC (EOC) tissues and exosomes [76]. The miR-200 family (including miR-200a, miR-200b, and miR-141) plays an important role in the EMT [179] and may be associated with a poorer prognosis in patients with EOC [180].

MiR-223 plays a critical role in regulating immune cell functions, including macrophage polarization and inflammatory responses [181]. This miRNA is also involved in the development and invasion of breast cancer and its resistance to treatment [182]. In the research conducted by Yang et al. [183], miR-223 levels in plasma exosomes from 78 patients with EOC, 40 patients with benign ovarian tumors, and 52 healthy controls were analyzed. Significantly higher levels of exosomal miR-223 were found in patients with EOC compared to healthy individuals and those with benign tumors. Higher expression of miR-223 was also observed in patients with metastases, and a correlation between CA125 and miR-223 levels suggested a link with disease progression [183]. Similar results of increased miR-223-3p expression were reported in the study by Fang et al. [184].

Further research focused on exosomal miRNA-205 [185], which is linked to the growth and progression of OC [176, 186]. A study involving 99 participants, including 36 with OC, 31 with benign tumors, and 32 healthy controls, showed higher expressions of miR-205 and CA125 in the OC groups compared to the others [185]. Higher levels of miR-205 were also noted in patients with LNMs, indicating that miR-205 is associated with OC and metastasis and may serve as a prognostic marker in patients with OC [185].

### Cervical cancer

The primary factor implicated in CC is the HPV [165]. Despite the availability of vaccines, the disease remains a significant health threat [187]. The progression of cervical cancer may be influenced by the dysregulation of exosomal miRNA [87]. miRNA-423-3p, known for its role in the development of cancers such as colorectal and lung cancer, also affects the malignancy of CC. Exosomes can affect the polarization of macrophages; TAMs are induced to become either M1 or M2 within the TME. M1 macrophages secrete pro-inflammatory cytokines to counteract cancer, while M2 macrophages produce cytokines that support cancer development [87].

In research conducted by Yan et al. [87] on CC cell lines HeLa, CaSki, and SiHa, as well as normal human CC lines, showed that patients with CC exhibited lower plasma exosomal miRNA expression (including miR-328–3p, miR-423–3p, miR-323a-3p, miR-10b-5p, miR-10a-5p, miR-125a-5p, miR-99b-5p, and miR-139–5p). There was a noted reduction in the expression of miRNA-423-3p in CC cell lines. It was also confirmed that exosomal miRNA-423-3p inhibits the M2 polarization of macrophages, leading to reduced malignancy of CC cells and tumor growth. This suggests that this miRNA could serve as a biomarker for CC treatment [87].

Other researchers have shown that the oncogenic lncRNA DLX6-AS1 plays a role in various cancers, including breast [188], bladder [189], and gastric cancer [190]. In additional studies involving 114 patients with CC, 60 individuals with cervical intraepithelial neoplasia (CIN), and 110 healthy controls, the role of exosomal lncRNAs in the progression and initiation of cervical cancer was examined [191]. Elevated levels of exosomal lncRNA DLX6-AS1 were found in the serum of CC patients compared to those with CIN and healthy individuals. A correlation between disease metastasis to lymph nodes and high expression of lncRNA DLX6-AS1 was also demonstrated. The researchers identify lncRNA DLX6-AS1 as a biomarker in the diagnosis of CC [191].

Other studies also confirm the role of lncRNA in the progression of CC [192]. Research on the role of lncRNA LINC01305 in the development of this cancer was conducted using 114 tissue samples from patients with CC. It was demonstrated that exosomes are the primary distributors of this RNA, which enhances cancer progression. Furthermore, a correlation was established between the level of expression and the survival rate of the patients studied. Higher expression in exosomes is associated with the progression of the disease [192].

### Vulvar cancer

Vulvar cancer (VC), which accounts for only 5% of gynecological cancers, occurs in four different histological types: squamous cell, basal cell, extramammary Paget disease, and melanoma. In the case of this cancer, the patient may not show symptoms for a long time, which results in delayed diagnosis and initiation of treatment procedures [193]. If the disease is detected quickly and treated effectively, patient survival can reach 88% [194].

Researchers’ attention is once again focusing on exosomal miRNAs due to the possibility of using them as biomarkers allowing the identification of tissues with cancer lesions. Changes in the expression of miRNAs in exosomal form (miR-21, miR-141, miR-200a, miR200b, miR-200c, miR-203, miR-205, miR-214) have been proven, regardless of their origin. This shows that the mentioned exosomes can be used identically to the miRNA profile in cancer tissue [180, 195].

Exosomal secretion is observed between tumor cells and the tumor environment. Rapid identification of this type of molecular transmitter can potentially be used to improve the diagnosis of gynecological cancers [196, 197]. However, the impact of exosomal particles is much broader due to their impact on the immune system, which actually affects the body’s homeostasis during cancer [196, 198]. It should be remembered that the described relationships do not occur individually but constitute a certain part of larger gene and protein relationships. The observed changes are therefore not the result of one factor but a complex process. For this reason, the rapid identification of exosomes as biomarkers specific to a given ailment may allow not only the early detection of cancer but also the determination of its stage, which is crucial when starting treatment of the patient [199].

## Limitations

Exosomal ncRNAs, including microRNAs, lncRNAs, and circular RNAs, have become key players in the regulation of gene expression and cellular processes [200]. In the case of reproductive system cancers, such as OC, EC, CC, and PCa, and exosomal ncRNAs have gained considerable attention as potential biomarkers and prognostic indicators due to their stability in body fluids and their ability to reflect the molecular state of tumors [201–204]. This area of ​​research holds promise for improving cancer diagnosis, prognosis, and treatment strategies.

The tumor environment is rich in a variety of cell types that secrete exosomes. Cancer cells secrete exosomes rich in oncogenic factors, exosomes derived from cancer-associated fibroblasts remodel the ECM and enhance progression, and immune cells carry exosomes that stimulate immune responses. In turn, platelet-derived exosomes have been shown to promote cancer cell survival [83]. Extracellular vesicles can be secreted by every cell in the body [205], which may implicate research due to the chaotic mix of exosomes in the blood. Exosomes carry a molecular fingerprint of the cell from which they were released [206]. A significant challenge in exosome identification is their internal cargo heterogeneity. This makes analysis in body fluids difficult, and the detection of a single exosome carrying a specific cargo may contribute to better diagnostics and faster cancer detection [207].

Future research directions should focus on (a) developing diagnostic biomarkers aimed at early detection of cancer and assessment of disease stage, as well as predicting treatment response; (b) elucidating the possible mechanism of their impact on the tumor microenvironment through in-depth studies on the ability to tumorigenesis and inhibit immune response; and (c) the therapeutic potential of exosomal ncRNAs. Currently, the possibility of using exosomes to assess the response to treatment in cancer diseases is being investigated. The challenge is how to determine the origin of exosomes because cells from the TME can secrete them and which can differ in size and cargo. In addition, the use of exosomes to transport specific contents may be an undoubtedly promising technique in cancer treatment [208]. From this perspective, further research is needed.

## Conclusion

Exosomes significantly influence the development and progression of cancer by facilitating communication between cancer cells and the microenvironment, as well as changing immune responses and increasing the possibility of metastasis. The review highlights the importance of exosomes as carriers of genetic markers that have the potential to be used in cancer diagnosis and treatment.

Moreover, the involvement of exosomes in reproductive system cancers highlights their role in both promoting and potentially inhibiting cancer through their complex interactions with cellular processes. As the incidence of cancer continues to increase worldwide, understanding the multifaceted role of exosomes offers promising avenues for developing new therapeutic strategies and improving patient outcomes. Future research should aim to elucidate the detailed mechanisms by which exosomes influence cancer progression and explore their potential for clinical applications, including more precise biomarkers and targeted delivery systems for cancer therapy.
